# Plasma miR-22-5p, miR-132-5p, and miR-150-3p Are Associated with Acute Myocardial Infarction

**DOI:** 10.1155/2019/5012648

**Published:** 2019-04-24

**Authors:** Huixian Li, Pengxiang Zhang, Fangjiang Li, Guili Yuan, Xiaoyuan Wang, Aiai Zhang, Feixing Li

**Affiliations:** Department of Cardiology, The First Affiliated Hospital of Hebei North University, Zhangjiakou City, Hebei Province 075000, China

## Abstract

Circulating microRNAs (miRNAs) are potential biomarkers for cardiovascular diseases. Our study aimed to determine whether miR-22-5p, miR-132-5p, and miR-150-3p represent novel biomarkers for acute myocardial infarction (AMI). Plasma samples were isolated from 35 AMI patients and 55 matched controls. Total RNA was extracted, and quantitative real-time PCR and ELISA were performed to investigate the expressions of miRNAs and cardiac troponin I (cTnI), respectively. We found that plasma levels of miR-22-5p and miR-150-3p were significantly higher during the early stage of AMI and their expression levels peaked earlier than cTnI. Conversely, circulating miR-132-5p was sustained at a low level during the early phase of AMI. All three circulating miRNAs were correlated with plasma cTnI levels. A receiver operating characteristic (ROC) analysis suggested that each single miRNA had considerable diagnostic efficacy for AMI. Moreover, combining the three miRNAs improved their diagnostic efficacy. Furthermore, neither heparin nor medications for coronary heart disease (CHD) affected plasma levels of miR-22-5p and miR-132-5p, but circulating miR-150-3p was downregulated by medications for CHD. We concluded that plasma miR-22-5p, miR-132-5p, and miR-150-3p may serve as candidate diagnostic biomarkers for early diagnosis of AMI. Moreover, a panel consisting of these three miRNAs may achieve a higher diagnostic value.

## 1. Introduction

Acute myocardial infarction (AMI) is one of the leading causes of death worldwide [1]. Diagnosing AMI early and accurately facilitates the immediate initiation of reperfusion therapy, which potentially reduces its mortality rate. Some biomarkers, such as creatine kinase-MB (CK-MB) and cardiac troponins (cTnI/cTnT), have been widely reported to be gold-standard indicators for the clinical diagnosis of AMI [2]. However, novel biomarkers, including molecular and genetic biomarkers, are being investigated for their ability to improve diagnostic accuracy and provide more prognostic information relevant to AMI [3].

MicroRNAs (miRNAs or miRs) are endogenous, small (19-25 nucleotides) single-stranded, noncoding RNAs that play a critical role in regulating gene expression by binding to the 3′ untranslated regions (3′-UTR) of target gene messenger RNAs (mRNAs) [4–6]. Numerous miRNAs have been demonstrated to play important roles in cell biological and pathological processes, including proliferation, differentiation, and apoptosis [7]. Notably, miRNAs are stable in various biological fluids, including serum, plasma, and urine. Reports have suggested that it may actually be possible to stabilize miRNAs under extreme conditions, including boiling, high or low pH, or extended storage and freeze-thaw cycles, by inducing the formation of protein-miRNA or chemical-miRNA complexes [8]. An increasing amount of evidence has shown that circulating miRNAs (e.g., miR-1, miR-133a, miR-499, and miR-208b) have the potential to serve as alternative biomarkers for the diagnosis of AMI [9, 10].

As a cardiac- and skeletal muscle-enriched miRNA, miR-22 has been reported to participate in cardiomyocyte hypertrophy and cardiac remodeling in response to stress [11]. The miR-132/212 cluster has been confirmed to play a critical role in cardiac hypertrophy [12]. As a dominant member of the miR-132/212 family, miR-132 negatively regulates cardiac function during cardiac remodeling by inducing angiogenesis. miR-150, a miRNA that is closely associated with inflammation, has been implicated in the pathogenesis of various cardiovascular diseases [13–15]. miR-150 has been shown to regulate high glucose-induced cardiomyocyte hypertrophy by targeting the transcriptional coactivator p300 [16]. Overall, these studies indicate that all three miRNAs play important roles in various cardiovascular diseases. Interestingly, a recent microarray analysis showed that the expression levels of miR-22, miR-132, and miR-150 were obviously dysregulated in patients with AMI [10]. However, the clinical significance of circulating miR-22-5p, miR-132-5p, and miR-150-3p in patients with AMI remains unclear. In this study, we investigated the dynamic expressions of miR-22-5p, miR-132-5p, and miR-150-3p in plasma obtained from patients in the early phase of AMI and explored the potential diagnostic value of these markers. Our findings suggest that circulating miR-22-5p, miR-132-5p, and miR-150-3p may be promising biomarkers for the early stage of AMI without affected by the affected by heparin or cardiovascular drugs. Furthermore, a composite of these miRNAs may provide superior predictive value.

## 2. Results

### 2.1. Patient Characteristics

**Figure 1:**
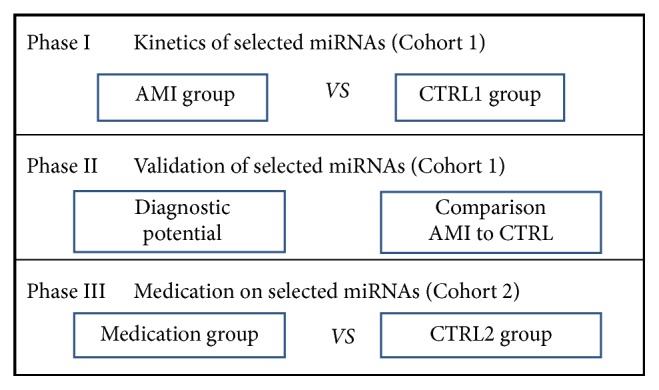
A flow chart of the study design strategy. The present study was conducted in the three following phases: (i) initial determination phase: we evaluated the dynamic expressions of the selected miRNAs in plasma obtained from patients with acute myocardial infarction (AMI) and control subjects (CTRL1), (ii) validation phase: we evaluated the potential diagnostic value of the selected miRNAs for discriminating AMI patients from controls, and (iii) determination phase: we evaluated the effects of heparin and medications on the plasma levels of selected miRNAs. CRTL1, control subjects presented with chest pain without coronary artery diseases (CHD); CRTL2, paroxysmal supraventricular tachycardia (PSVT) patients without medications for CHD; Medication, patients taking medications for CHD.

Two cohorts comprising 180 participants were enrolled in the present study (Figure 1). In the first cohort, we enrolled and assessed the clinical characteristics of 35 AMI patients and 55 control subjects. These results are listed in Table 1. There were no significant differences between the control subjects and the AMI patients for most of the considered clinical variables, including total cholesterol (TC), total triglycerides (TG), high-density lipoprotein (HDL), low-density lipoprotein (LDL), systolic/diastolic blood pressure (SBP/DBP), body mass index (BMI), creatinine (Cr), history of diabetes, and smoking status. However, white blood cell counts and CK-MB were significantly higher in the AMI patients than in the control subjects, which is in agreement with previous studies. Additionally, the medications that were used to treat AMI were significantly different between the AMI patients and control subjects.

Subsequently, we explored the potential effects of medications for coronary heart disease (CHD) on the expressions of selected miRNAs in the second cohort (40 patients with CHD using medication and 50 patients with paroxysmal supraventricular tachycardia). There were significant differences between the two groups in the following characteristics: history of hypertension or hyperlipidemia and related medications (Table 2).

### 2.2. Expression Patterns and the Correlation of Plasma miRNAs and cTnI

**Figure 2:**
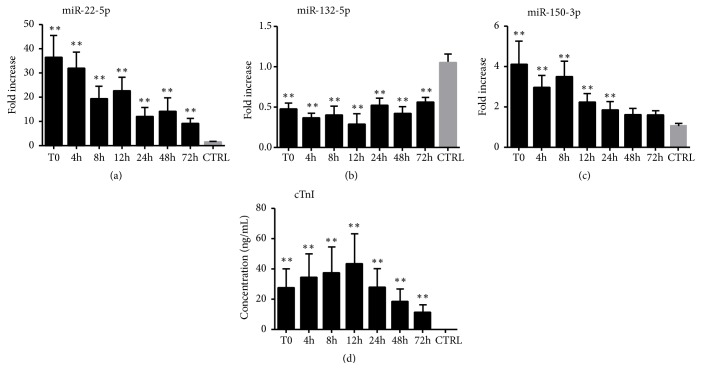
The plasma levels of miRNAs (quantitative real-time PCR) and cTnI (immunoassay) in the AMI and control groups. The plasma levels of miR-22-5p (a), miR-132-5p (b), miR-150-3p (c), and cTnI (d) in the AMI and control groups. AMI, patients with acute myocardial infarction; CRTL, control subjects; T0, the initial blood sample collection time after the onset of AMI symptoms. Data are shown as the mean ± SD, *∗*p<0.05, *∗∗*p<0.01 versus CTRL. AMI, patients with acute myocardial infarction, n=35; CRTL, control subjects, n=55; cardiac troponin I (cTnI).

To investigate the expression patterns of selected miRNAs (miR-22-5p, miR-132-5p, and miR-150-3p) and cTnI during the early phase of AMI, we used quantitative real-time PCR (qRT-PCR) and enzyme-linked immunosorbent assay (ELISA) to measure the expression levels of these miRNAs and cTnI in plasma obtained from AMI patients and control subjects. Overall, the expression levels of circulating miR-22-5p and miR-150-3p were significantly higher in the AMI patients than in the control subjects, whereas miR-132-5p presented the opposite trend (Table 3). Compared to the controls, the expression of plasma miR-22-5p in the AMI patients increased and reached the peak at T0 (36.47 fold) and then gradually decreased but still upregulated till 72 h after T0 (Figure 2(a)). Meanwhile, plasma miR-150-3p also upregulated and achieved peak at T0 (4.09 fold), but the expression level quickly decreased and return to normal at 48 h after T0 (Figure 2(c)). In contrast, circulating miR-132-5p in plasma exhibited a sustained low level of expression at all time-points in the early stage of AMI of AMI patients compared to the control subjects (Figure 2(b)). Plasma cTnI levels were also measured in the same samples, which showed that the expression levels of cTnI were higher in the AMI patients at T0, achieved peak at 12 h (2518 fold), and then gradually decreased but still upregulated till 72 h compared to the control subjects (Figure 2(d)) (Table 3).

**Figure 3:**
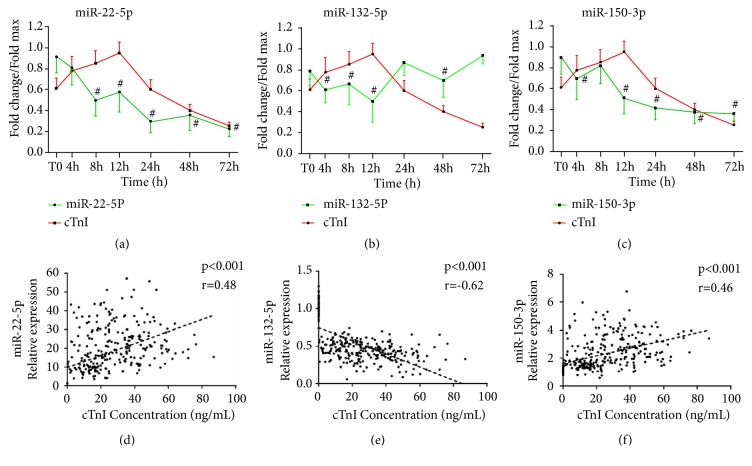
The dynamic expressions of plasma miRNAs (quantitative real-time PCR) and cTnI (immunoassay) in the AMI group and the correlations between them. (a) The expression patterns of plasma miR-22-5p and cTnI in the AMI group; (b) the expression patterns of plasma miR-132-5p and cTnI in the AMI group; and (c) the expression patterns of plasma miR-150-3p and cTnI in the AMI group. (d) The correlation between plasma levels of miR-22-5p and cTnI. (e) The correlation between plasma levels of miR-132-5p and cTnI. (f) The correlation between plasma levels of miR-150-3p and cTnI. Data are shown as the mean ± SD, #p<0.05 versus peak level in the AMI group. AMI, patients with acute myocardial infarction, n=35; CRTL, control subjects, n=55; cardiac troponin I (cTnI).

Firstly, we found that the relative expression of cTnI reached its peak at 12 h after T0, which was later than the peaks for miR-22-5p and miR-132-5p (Figures 3(a)–3(c)). Meanwhile, the result showed that circulating miR-22-5p (r = 0.48, p<0.01) and miR-150-3p (r = 0.46, p<0.01) levels were both positively correlated with cTnI levels. In contrast, plasma miR-132-5p and cTnI were negatively correlated (r = -0.62, p<0.01) (Figures 3(d)–3(f)).

### 2.3. Diagnostic Efficiency of miRNAs in the Early Stage of AMI

To conveniently evaluate the power of the selected miRNAs to predict AMI, we converted the expression levels of these miRNAs into single score or combined score and then performed a receiver operating characteristic (ROC) analysis, as previously described [17, 18].

**Figure 4:**
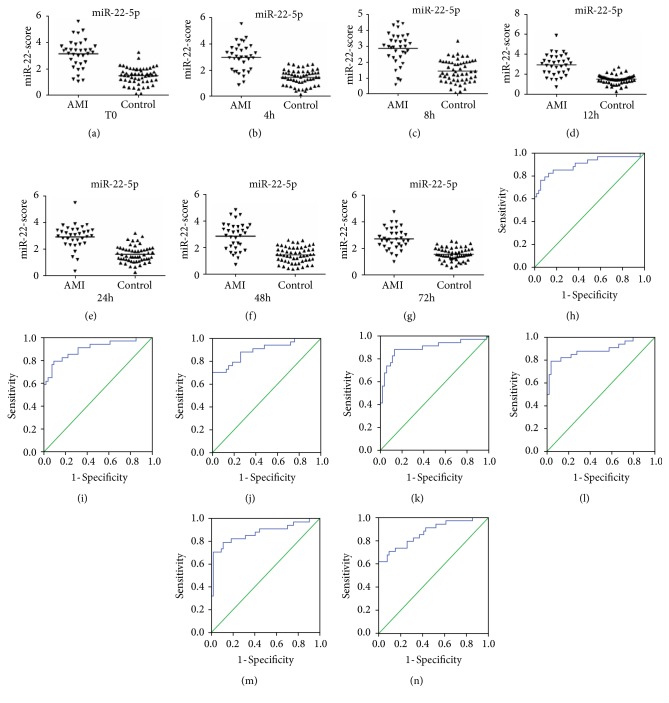
Discriminatory power of plasma miR-22-5p scores. The miR-22-5p scores are presented as the mean values in the AMI and control groups at T0 (a), 4 h (b), 8 h (c), 12 h (d), 24 h (e), 48 h (f), and 72 h (g). Receiver operator characteristic (ROC) curves for miR-22-5p at T0 (h), 4 h (i), 8 h (j), 12 h (k), 24 h (l), 48 h (m), and 72 h (n). AMI, patients with acute myocardial infarction, n=35; CRTL, control subjects, n=55.

**Figure 5:**
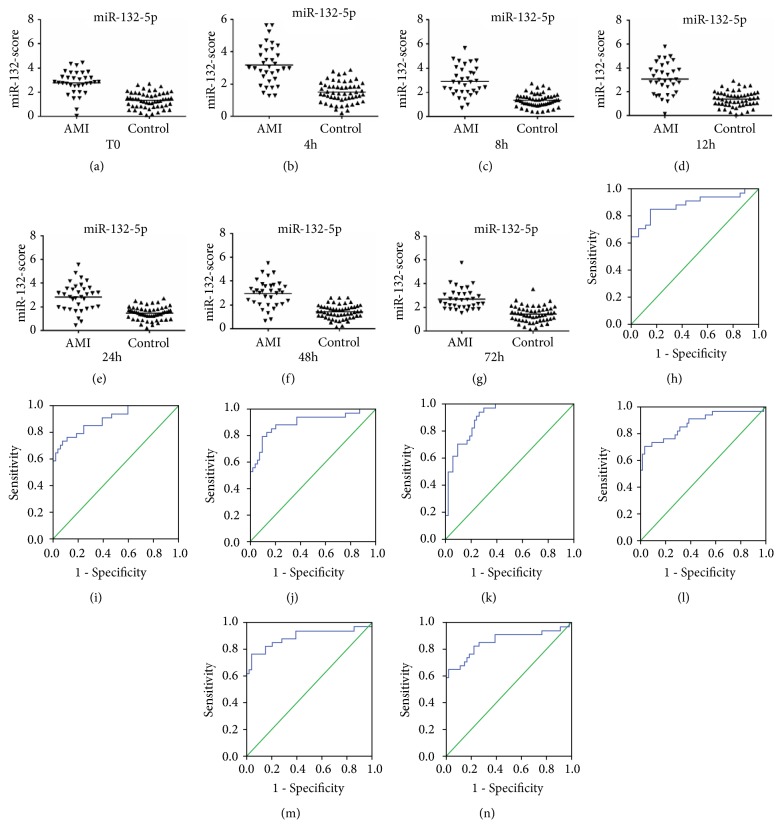
Discriminatory power of plasma miR-132-5p scores. The miR-132-5p scores are presented as the mean values for the AMI and control groups at T0 (a), 4 h (b), 8 h (c), 12 h (d), 24 h (e), 48 h (f), and 72 h (g). Receiver operator characteristic (ROC) curves for miR-132-5p at T0 (h), 4 h (i), 8 h (j), 12 h (k), 24 h (l), 48 h (m), and 72 h (n). AMI, patients with acute myocardial infarction, n=35; CRTL, control subjects, n=55.

**Figure 6:**
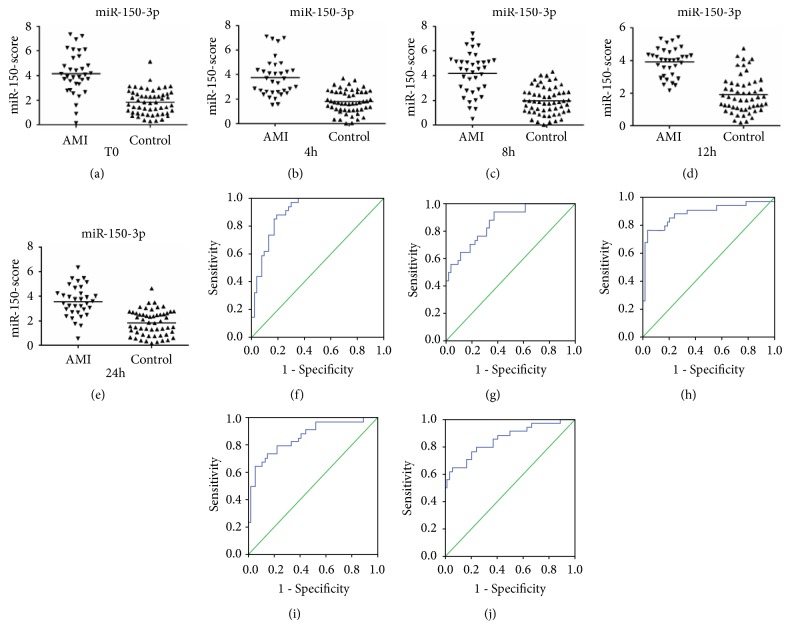
Discriminatory power of plasma miR-150-3p scores. The miR-150-3p scores are presented as the mean values for the AMI and control groups at T0 (a), 4 h (b), 8 h (c), 12 h (d), and 24 h (e). Receiver operator characteristic (ROC) curves for miR-150-3p at T0 (f), 4 h (g), 8 h (h), 12 h (i), and 24 h (j). AMI, patients with acute myocardial infarction, n=35; CRTL, control subjects, n=55.

**Figure 7:**
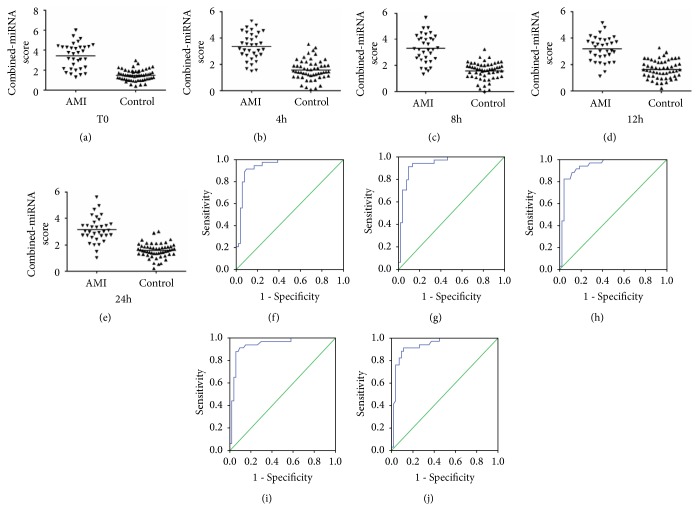
Discriminatory power of combined-miRNA scores. The combined-miRNA scores are presented as the mean values for the AMI and control groups at T0 (a), 4 h (b), 8 h (c), 12 h (d), and 24 h (e). Receiver operator characteristic (ROC) curves for miR-150-3p at T0 (f), 4 h (g), 8 h (h), 12 h (i), and 24 h (j). The combined-miRNA scores include the scores for miR-22-5p, miR-132-5p, and miR-150-3p. AMI, patients with acute myocardial infarction, n=35; CRTL, control subjects, n=55.

The mean scores for miR-22-5p were 3.13 (T0), 2.99 (4 h), 2.88 (8 h), 2.94 (12 h), 2.93 (24 h), 2.88 (48 h), and 2.70 (72 h) in the AMI group and 1.49, 1.48, 1.45, 1.51, 1.61, 1.47, and 1.54, respectively, in the control group (Figures 4(a)–4(g)). The mean scores for miR-132-5p were 2.78 (T0), 3.18 (4 h), 2.92 (8 h), 3.08 (12 h), 2.85 (24 h), 2.96 (48 h), and 2.71 (72 h) in the AMI group and 1.34, 1.50, 1.35, 1.38, 1.48, 1.43, and 1.44, respectively, in the control group (Figures 5(a)–5(g)). The mean scores for miR-150-3p were 4.15 (T0), 3.75 (4 h), 3.95 (8 h), 3.71 (12 h), and 3.58 (24 h) in the AMI group and 1.86, 1.80, 1.91, 1.92, and 1.84, respectively, in the control group (Figures 6(a)–6(e)). However, when the three circulating miRNAs were combined, the mean scores for the combined miRNAs were 3.24 (T0), 3.19 (4 h), 3.17 (8 h), 3.20 (12 h), and 3.01 (24 h) in the AMI group and 1.72, 1.74, 1.71, 1.74, and 1.79, respectively, in the control group (Figures 7(a)–7(e)).

The ROC curve analysis indicated that miR-22-5p, miR-132-5p, and miR-150-3p (Figures 4(h)–4(n), 5(h)–5(n), and 6(f)–6(j)) separately showed a moderate power (sensitivity and specificity) for distinguishing value for the AMI during the early phase of chest pain. However, when these three miRNA were combined, A panel of these miRNAs presented higher AUC values than the single one (Figures 7(f)–7(j)) (Table 4).

### 2.4. The Impact of Heparin and Medications on miRNAs Expression

**Figure 8:**
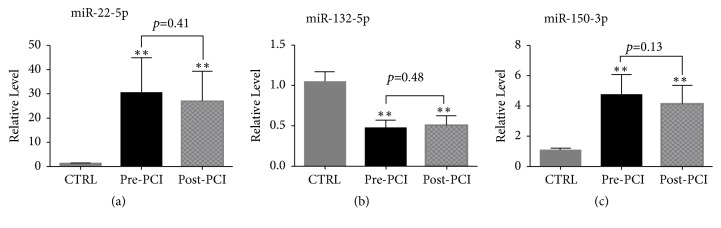
The impact of heparin on plasma miRNA (Quantitative real-time PCR) expression levels during the PCI procedure. The expression levels of circulating miR-22-5p (a), miR-132-5p (b), and miR-150-3p (c) in the pre-PCI and post-PCI plasma of AMI patients. AMI, patients with acute myocardial infarction; PCI, percutaneous coronary intervention; CRTL, control subjects. Data are shown as the mean ± SD, *∗∗*p<0.01 versus CTRL. Pre-PCI, previous-percutaneous coronary intervention, n=35; Post-PCI, postprocedure percutaneous coronary intervention, n=35; CRTL, control subjects, n=55.

It has been reported that heparin and medications might affect the expression of some circulating miRNAs, potentially limiting the diagnostic reliability of affected miRNAs [19]. Hence, we firstly investigated whether heparin impacted the levels of the selected miRNAs during the PCI procedure in AMI patients. The results showed that there were no significant differences between pre-PCI and post-PCI plasma samples in the expression levels of the three miRNAs, suggesting that heparin did not impact the expression of these selected miRNAs (Figure 8).

**Figure 9:**
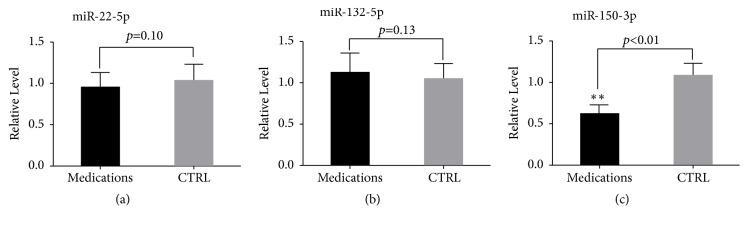
The impact of CHD medications on plasma miRNA expression levels. The expression of plasma miR-22-5p (a), miR-132-5p (b), and miR-150-3p (c) in the medication and control groups. Medications, patients taking medications, n=40; CRTL, patients without medications, n=50. Data are shown as the mean ± SD; *∗∗p*<0.01* versus* CTRL. Coronary heart disease CHD.

In the initial phase of the present study, we observed that medications (e.g., angiotensin converting enzyme inhibitors (ACEI), beta-blockers, nitrates, statins, aspirin, or clopidogrel) used to treat AMI were significantly different between the patients with AMI and the control subjects. Consequently, to determine whether these medications affect plasma miRNAs expression, we enrolled an additional well-matched cohort in which we analyzed the expression levels of miR-22-5p, miR-132-5p, and miR-150-3p. However, only circulating miR-150-3p was significantly different between the two groups, indicating that these medications may reduce the expression of plasma miR-150-3p and thereby be partially involved in its early regression to baseline levels (Figure 9).

## 3. Discussion

In the present study, we provided evidence that circulating miR-22-5p, miR-132-5p, and miR-150-3p might serve as novel promising diagnostic biomarkers for the early diagnosis of AMI. We showed that plasma miR-22-5p and miR-150-3p levels were markedly elevated in the early phase of AMI and that they reached peak expression earlier than cTnI, whereas the expression of miR-132-5p displayed a sustained downward trend during the early phase of AMI. Furthermore, we demonstrated that miR-22-5p, miR-132-5p, and miR-150-3p each individually showed a moderate ability to discriminate AMI patients from controls, while a multi-miRNA approach presented higher discriminatory power than any single miRNA. Additionally, we proved that the expressions of these miRNAs were not influenced by heparin, whereas medications for CHD may reduce the expression of plasma miR-150-3p during the early stage of AMI. There is no doubt that cTnI has been widely used as indicators for the clinical early diagnosis of AMI, however which may be unable to reliably rule in or rule out AMI immediately on admission for early stage of patients with chest pain. Our investigation showed that selected circulating miRNA had an earlier upregulation and higher diagnostic accuracy for AMI, and a combined miRNAs might sever as excellent potential early diagnostic biomarker for AMI in future.

A large number of studies have shown that miRNAs are implicated in the pathogenesis of various cardiovascular diseases or disorders [20–22]. Moreover, accumulating evidence has indicated that circulating miRNAs, including heart-, vascular-, and endothelium-enriched miRNAs, might serve as potential biomarkers for various cardiovascular diseases, including AMI, unstable angina pectoris (UAP), atrial fibrillation acute myocarditis, heart failure, and acute pulmonary embolism [19, 23–27]. So far, most published studies investigating the use of miRNAs as biomarkers for AMI have focused mainly on myocardium-derived miRNAs [3, 10]. However, a variety of physiological and pathological changes occur during the AMI process, including disordered endothelial function, plaque erosion/rupture, platelet aggregation, oxidative stress, inflammatory reactions, and myocardial cell injury/necrosis [18, 28]. A recent study by Wang et al. [18] showed that miRNAs that originate from various cell types during the early course of AMI might sever as novel biomarkers for AMI.

It was previously reported that all of the miRNAs selected for this study are involved in the functional regulation of cardiovascular-derived cells and cardiovascular diseases. As a cardiac-abundant miRNA, miR-22 is critically involved in stress-induced cardiac hypertrophy and remodeling [11]. It has been reported to induce cardiac hypertrophy, in part by targeting PTEN on cardiomyocytes [29]. Yang J.* et al*. found that miR-22 also protects cardiomyocytes from apoptosis by decreasing the expression of CBP/p300 and acetylated p53 [30]. Additionally, circulating miR-22 has been used as a potential predictive marker for several diseases, including chronic nervous lesions, osteosarcoma and aortic stenosis [31, 32]. As an endothelial cell-specific miRNA, miR-132 plays crucial roles in the regulation of angiogenesis-stimulating factors and the angiogenic process by directly targeting Rasa1, Spred1, and p120RasGAP [12, 33, 34]. Eskildsen T. V.* et al*. found that miR-132 plays a critical role in regulating cardiovascular functions, such as cardiac hypertrophy, heart failure and blood pressure, by targeting AT1R [35]. miR-150, an inflammation-associated miRNA, was also reported to be involved in AMI-induced injury by regulating monocyte migration and the production of proinflammatory cytokines [36]. Additionally, circulating miR-150 was reported to be associated with left ventricular contractility post-MI [37]. Moreover, previous studies have shown that circulating miR-150 may be a novel biomarker for platelet activation and disorders in patients with UAP [38]. Interestingly, in the present study, we found that plasma miR-150-3p levels rapidly declined during the early phase of AMI, and we showed that medications may contribute to its early reduction. However, we did not identify a specific medication that was responsible for this effect. A previously study [39] reported a significant downregulation of plasma miR-150 following antiplatelet therapy, suggesting that aspirin and/or clopidogrel might be responsible for the gradual reduction we observed in plasma miR-150-3p levels.

There are also some limitations to the present study. First, future studies including larger populations will be needed to confirm the clinical usefulness of the selected miRNAs in diagnosing AMI. Second, although the expression peaks for miR-22-5p and miR-150-3p occurred earlier than that of cTnI, future studies should determine whether the initial increases in circulating miR-22-5p and miR-150-3p occurs earlier than cTnI. Furthermore, additional studies are required to investigate the mechanisms underlying the dysregulation of these miRNAs and to address whether they are mechanistically involved in the pathophysiological processes underlying AMI.

In conclusion, we have performed the first investigation to examine the dynamic expressions of circulating miR-22-5p, miR-132-5p, and miR-150-3p during the early phase of AMI. Our findings indicate that plasma miR-22-5p, miR-132-5p, and miR-150-3p may be considered novel and validated biomarkers for AMI diagnosis. Moreover, a combined panel consisting of these three miRNAs may provide greater diagnostic value for the early identification of AMI.

## 4. Materials and Methods

### 4.1. Study Design and Ethical Statement

The study protocol was conducted according to the principles expressed in the Declaration of Helsinki and approved by the Medical Ethics Committee of the First Affiliated Hospital of Hebei North University. All participants were inpatients admitted to the Department of Cardiology of First Affiliated Hospital of Hebei North University between December 2015 and August 2017. Written informed consent was obtained from all the involved participants.

In the first cohort, we enrolled 35 consecutive AMI patients and 55 control subjects (CTRL1 group) who presented with chest pain without CHD. The inclusion criteria for AMI patients were based on the most recently developed universal definition of myocardial infarction (MI) [40]. All AMI patients were diagnosed with AMI for the first time and were successfully treated using PCI. A coronary angiography (CAG) or coronary computer tomography angiography (CTA) was performed on each of the control subjects to exclude CHD. Clinical histories and medication records were collected from all participants. The exclusion criteria for all patients were the following: a previous history of impaired left ventricular ejection fraction of ≤ 45%, congestive heart failure, known malignancy, chronic kidney or hepatic diseases, and surgery or skeletal muscle damage within the previous months that would affect the expression of miRNAs.

In the second cohort, we enrolled 40 patients with a history of hypertension and hyperlipidemia who had been on medications for CHD (e.g., ACEI, beta-blockers, nitrates, statins, aspirin, or clopidogrel) for at least 2 weeks into the medication group, and we enrolled 50 age- and gender-matched patients with paroxysmal supraventricular tachycardia (PSVT) who had not taken any of these medications in the past two weeks into the control group (CTRL2 group). We then investigated whether these medications affected plasma miRNA expression. The exclusion criteria for the second cohort were the same as those used for the first cohort.

### 4.2. Blood Sample Collection and Storage

All peripheral venous blood samples were obtained from the AMI patients upon admission to Union hospital. The initial blood sample collection time (T0) was 9.24 ± 2.81 h after the onset of AMI symptoms. Subsequent blood samples were obtained at 4 h ± 30 min (4 h), 12 h ± 30 min (12 h), 24 h ± 30 min (24 h), 48 h ± 30 min (48 h), and 72 h ± 30 min (72 h) after T0. Additionally, plasma was collected pre- and post-PCI procedures to investigate whether heparin had an impact on the expression of selected miRNAs in plasma.

All samples (4-8 mL) were collected into K2-EDTA-coated tubes (BD, NJ, USA). The samples were stored for up to 24 h at 4°C and prepared according to the following 2-step centrifugation protocol: 1,500 x g for 15 min at 4°C, then 14,000 x g at 4°C for another 15 min. After centrifugation, the supernatant (plasma) was aliquoted into RNase/DNase-free tubes and stored at −80°C for subsequent experiments.

### 4.3. Isolation of Circulating RNA from Plasma

Total RNA was extracted from frozen plasma using TRIzol Reagent BD (TR126, MRC, Cincinnati, OH, USA) according to the manufacturer's instructions. Briefly, 250 *μ*L of plasma was mixed with 750 *μ*L of TRIzol (1:3, v/v) and incubated for 15 min at room temperature to ensure the complete dissociation of nucleoprotein complexes. After 200 *μ*L of chloroform was added, the mixture was vortexed vigorously for 15 sec. After extraction was performed for 15 min at room temperature, the mixture was centrifuged at 12,000 x g for 15 min at 4°C. The upper aqueous phase was transferred to fresh reagent tubes, an equal volume of cold isopropanol was added, and the mixture was stored at −20°C overnight. The samples were centrifuged at 12,000 x g for 15 min at 4°C. The supernatant was then removed, and the RNA pellet was washed with 1 mL of 75% (v/v) ethanol to remove residual salt. For normalization, each sample was supplemented with 25 pM* Caenorhabditis elegans* miR-39 (cel-miR-39) after the TRIzol was added, as previously reported [41].

## 5. MicroRNA Analysis

Total RNA (1 *μ*g) was reverse-transcribed using a cDNA Reverse Transcription Kit (TAKARA, Shiga, Japan) according to the manufacturer's protocol. Briefly, the mixed reagents were incubated for 60 min at 42°C, 10 min at 70°C, and then 4°C for 5 min. qRT-PCR was performed using cel-miR-39 as the normalization control with a Bulge-Loop™ miRNA RT-qPCR Detection Kit (Ribobio, Guangzhou, China) and SYBR Green PCR Master Mix Kit (TAKARA, Shiga, Japan). Briefly, the reactions were incubated at 95°C for 20 s, followed by 40 cycles of 95°C for 10 s, 60°C for 20 s, and 70°C for 1 s (a cycle threshold (Ct) ≥ 40 was considered to indicate undetermined). All data were analyzed using Bio-Rad CFX Manager software (Bio-Rad, CA, USA). The Ct values were normalized to cel-miR-39 using the formula 2^-(Ct  [miRNA]-Ct  [cel-miRNA-39])^, and the 2^-ΔΔCt^ method was used to analyze the relative expression levels of the miRNAs.

### 5.1. Cardiac Troponin I Analysis

All plasma samples were stored at –80°C prior to analysis. All frozen plasma samples were thawed and deidentified for research and clinical purposes. Then, using an sandwich chemiluminescent magnetic microparticle immunoassay method, the Abbott–Architect Troponin I assay (Abbott Diagnostics, Abbott Park, IL, USA) was performed, with a limit of detection of 0.01 ng/ml, a 99th-percentile cutoff point of 0.028 ng/ml, and a coefficient of variation (CV) of less than 10% at 0.032 ng/ml, as specified by the manufacturer. The normal reference range for cTnI was established as < 0.03 ng/ml.

### 5.2. Statistical Methods

All data were presented as the mean ± standard deviation (SD). Student's two-sided t-tests or one-way ANOVA followed by Bonferroni's multiple comparison tests (as a post hoc analysis) were used for normally distributed values. For variables without a normal distribution, the Kruskal–Wallis and Mann–Whitney tests were performed. The time-courses of the expression levels of the miRNAs and cTnI were analyzed using repeated-measures ANOVA. For categorical clinical variables, the Chi-Square (*χ*2) test (or Fisher's exact test when necessary) was used. The correlation analysis of miRNAs and cTnI was performed using a linear regression analysis. All statistical tests were two-tailed, and p < 0.05 was considered statistically significant.

The ROC curves for the plasma miRNAs were analyzed to discriminate AMI patients from control subjects, and the areas under the ROC curves (AUCs) were estimated to assess the diagnostic accuracy of the identified miRNAs. Herein, an inverted-normalized miRNA signal (the miRNA-score) was used to represent the relative expression level of the selected miRNAs in the AMI group in comparison to the control group. As previously reported [17, 18], the miRNA scores were calculated by deducting the normalized Ct from 40 and then adjusted by subtracting the minimal score (so that the miRNA scores started at 0). The optimal diagnostic points for each of the miRNAs were assessed at cutoff values using the largest Youden's index (sensitivity + specificity - 1). All statistical analyses were performed using SPSS 13.0 (Chicago, IL, USA).

## Figures and Tables

**Table 1:** Clinical characteristics of the AMI patients and control subjects in the first cohort.

Variable	AMI group	Control group	*p*-value
	(n = 35)	(n = 55)	
Male/Female (n/n)	15/20	27/28	0.26
Age (years)	60.86 ± 11.25	56.36 ± 12.36	0.09
Body mass index (kg/m2)	25.43 ± 2.82	24.52 ± 2.52	0.12
Smoking status			
Current smoker (%)	6 (17%)	4 (7%)	0.15
Former smoker (%)	12 (34%)	11 (20%)	0.13
Hypertension, n (%)	11 (31%)	12 (22%)	0.31
Hyperlipidaemia, n (%)	13 (37%)	14 (25%)	0.24
Diabetes mellitus, n (%)	5 (14%)	6 (11%)	0.63
WBC (x 10^9^ /L)	9.35 ± 1.05	5.84 ± 1.28	<0.01*∗∗*
SBP (mmHg)	129.01 ± 15.32	128.52 ± 10.69	0.09
DBP (mmHg)	74.21 ± 6.59	76.90 ± 7.87	0.10
Heart rate (beats/minutes)	75.42 ± 12.57	74.05 ± 12.05	0.61
TC (mmol/L)	4.84 ± 0.73	4.46 ± 1.09	0.07
HDL (mmol/L)	1.37 ± 0.17	1.39 ± 0.19	0.61
LDL (mmol/L)	3.13 ± 0.55	2.93 ± 0.68	0.15
TG (mmol/L)	1.64 ± 0.69	1.71 ± 0.75	0.66
Cr (umol/L)	74.27 ± 15.02	71.28 ± 10.96	0.28
CK-MB (IU/L)	195.67 ± 63.20	8.56 ± 1.87	<0.01*∗∗*
Medications			
ACE inhibitors (%)	22 (63%)	8 (15%)	<0.01*∗∗*
Beta-blockers (%)	27 (77%)	6 (11%)	<0.01*∗∗*
Nitrates (%)	25 (71%)	/	<0.01*∗∗*
Statins (%)	35 (100%)	14 (25%)	<0.01*∗∗*
Aspirin (%)	35 (100%)	8 (15%)	<0.01*∗∗*
Clopidogrel (%)	35 (100%)	/	<0.01*∗∗*

Data are shown as the mean ± SD, *∗p*<0.05, *∗∗p*<0.01* versus* control subjects.

**Table 2:** Clinical characteristics of the enrolled subjects in the second cohort.

Variable	Medication group	Control group	*p*-value
	(n = 40)	(n = 50)	
Male/Female (n/n)	17/23	20/30	0.24
Age (years)	50.86 ± 12.30	46.36 ± 10.25	0.06
Body mass index (kg/m^2^)	24.31 ± 2.34	23.74 ± 1.95	0.21
Smoking status			
Current smoker (%)	6 (15%)	4 (8%)	0.33
Former smoker (%)	7 (18%)	4 (8%)	0.17
Hypertension history, n (%)	17 (43%)	8 (16%)	<0.01*∗∗*
Hyperlipidaemia history, n (%)	25 (63%)	7 (14%)	<0.01*∗∗*
Diabetes mellitus, n (%)	4 (10%)	3 (6%)	0.70
WBC (x 10^9^ /L)	5.75 ± 1.15	5.64 ± 1.24	0.67
Heart rate (beats/minutes)	72.42 ± 10.86	75.05 ± 9.62	0.23
Cr (umol/L)	73.25 ± 14.38	69.72 ± 13.40	0.23
Medications			
ACE inhibitors (%)	14 (35%)	5 (10%)	<0.01*∗∗*
Beta-blockers (%)	11 (28%)	3 (6%)	<0.01*∗∗*
Nitrates (%)	5 (13%)	/	<0.01*∗∗*
Statins (%)	25 (63%)	7 (14%)	<0.01*∗∗*
Aspirin (%)	10 (25%)	3 (6%)	0.02*∗*
Clopidogrel (%)	6 (15%)	/	<0.01*∗∗*

Data are shown as the mean ± SD, *∗p*<0.05, *∗∗p*<0.01 patients for medications* versus* control subjects.

**Table 3:** The relative expression of plasma miRNAs and cTnI in early stage of AMI.

Fold change	AMI (0h)	AMI (4h)	AMI (8h)	AMI (12h)	AMI (24h)	AMI (48h)	AMI (72h)
miR-22-5p	36.47 ± 9.11*∗*	31.81 ± 6.89*∗*	19.37 ± 5.09*∗*	22.38 ± 5.89*∗*	11.71 ± 4.09*∗*	14.06 ± 5.75*∗*	8.72 ± 2.52*∗*
miR-132-5p	0.47 ± 0.08*∗*	0.36 ± 0.06*∗*	0.39 ± 0.12*∗*	0.29 ± 0.13*∗*	0.52 ± 0.09*∗*	0.42 ± 0.09*∗*	0.56 ± 0.06*∗*
miR-150-3p	4.09 ± 1.20*∗*	2.93 ± 0.64*∗*	3.48 ± 0.80*∗*	2.19 ± 0.47*∗*	1.84 ± 0.44*∗*	1.60 ± 0.32	1.57 ± 0.25
cTnI	1578 ± 769*∗*	2001 ± 940*∗*	2198 ± 1015*∗*	2518 ± 1200*∗*	1597 ± 767*∗*	1050 ± 518*∗*	654 ± 291*∗*

Data are shown as the mean ± SD, *∗p*<0.05, plasma expression of miR-22-5p, miR-132-5p, miR-150-3p, and cTnI at different time points in early stage of AMI* versus* control subjects.

**Table 4:** Diagnostic value of circulating selected-miRNAs for AMI.

Time point	miRNAs	AUC	95% CI	p value	Cut off value	Sensitivity	Specificity
T0	miR-22-5p	0.901	0.827-0.976	p<0.001	1.9837	0.882	0.759
	miR-132-5p	0.886	0.805-0.967	p<0.001	1.8314	0.853	0.741
	miR-150-3p	0.904	0.843-0.964	p<0.001	2.5457	0.882	0.759
	*Combined score*	0.942	0.893-0.991	p<0.001	2.2850	0.912	0.870
4h	miR-22-5p	0.901	0.831-0.972	p<0.001	1.8764	0.853	0.741
	miR-132-5p	0.900	0.833-0.967	p<0.001	1.8517	0.853	0.741
	miR-150-3p	0.867	0.793-0.941	p<0.001	2.5124	0.794	0.685
	*Combined score*	0.938	0.886-0.990	p<0.001	2.2085	0.941	0.842
8h	miR-22-5p	0.895	0.822-0.968	p<0.001	1.8569	0.853	0.741
	miR-132-5p	0.897	0.823-0.967	p<0.001	1.6987	0.882	0.759
	miR-150-3p	0.887	0.805-0.970	p<0.001	2.4592	0.882	0.759
	*Combined score*	0.943	0.894-0.992	p<0.001	2.1300	0.912	0.815
12h	miR-22-5p	0.895	0.815-0.975	p<0.001	2.0128	0.882	0.741
	miR-132-5p	0.909	0.851-0.967	p<0.001	1.7088	0.912	0.759
	miR-150-3p	0.859	0.778-0.941	p<0.001	2.6464	0.794	0.704
	*Combined score*	0.941	0.888-0.994	p<0.001	2.0900	0.912	0.852
24h	miR-22-5p	0.893	0.814-0.972	p<0.001	1.9837	0.853	0.741
	miR-132-5p	0.878	0.798-0.958	p<0.001	1.7683	0.824	0.704
	miR-150-3p	0.853	0.768-0.939	p<0.001	2.5121	0.794	0.704
	*Combined score*	0.936	0.883-0.989	p<0.001	2.1700	0.912	0.796
48 h	miR-22-5p	0.874	0.789-0.959	P=0.001	1.9946	0.824	0.722
	miR-132-5p	0.895	0.814-0.976	p<0.001	1.8083	0.853	0.741
72h	miR-22-5p	0.870	0.791-0.950	P=0.001	1.8744	0.794	0.704
	miR-132-5p	0.859	0.768-0.951	p<0.001	1.8334	0.853	0.722

Combined score, the combination of miR-22-5p, miR-132-5p, and miR-150-3p scores; AUC, area under the ROC curve; 95% Cl, 95% confidence interval.

## Data Availability

The data used to support the findings of this study are available from the corresponding author upon request.

## Abbreviations

ACEI: Angiotensin converting enzyme inhibitor
AMI: Acute myocardial infarction
AUC: Area under the ROC curve
BMI: Body mass index
CAG: Coronary angiography
CHD: Coronary heart disease
CI: Confidence interval
CK-MB: Creatine kinase-MB
Cr: Creatinine
cTnI/T: Cardiac troponin I/T
DBP: Diastolic blood pressure
HDL: High-density lipoprotein
LDL: Low-density lipoprotein
miRNAs or miRs: MicroRNAs
mRNAs: Messenger RNAs
PCI: Percutaneous coronary intervention
PSVT: Paroxysmal supraventricular tachycardia
qRT-PCR: Quantitative real-time PCR
ROC: Receiver operating characteristic
SBP: Systolic blood pressure
SD: Standard deviation
TC: Total cholesterol
TG: Total triglyceride
UAP: Unstable angina pectoris
WBC: White blood cell.
